# Current status and prospect of transcutaneous auricular vagus nerve stimulation for disorders of consciousness

**DOI:** 10.3389/fnins.2023.1274432

**Published:** 2024-01-08

**Authors:** Yifei Wang, Jinling Zhang, Weihang Zhai, Yu Wang, Shaoyuan Li, Yi Yang, Yanfeng Zheng, Jianghong He, Peijing Rong

**Affiliations:** ^1^Institute of Acupuncture and Moxibustion, China Academy of Chinese Medical Sciences, Beijing, China; ^2^Department of Neurosurgery, Beijing Tiantan Hospital, Capital Medical University, Beijing, China

**Keywords:** transcutaneous auricular vagus nerve stimulation, disorders of consciousness, neuromodulation, vagus nerve, treament

## Abstract

Disordered Consciousness (DOC) is among neurological disorders for which there is currently no admitted treatment. The pathogenesis of DOC is still unclear, covering a variety of indistinguishable types of diseases, high misdiagnosis rate and poor prognosis. Most treatments remain to be clarified in the future to provide adequate evidence for clinical guidance. Neuromodulation technology aims to regulate neural circuits to promote awakening more directly. At present, it is confirmed that the potential of transcutaneous auricular vagus nerve stimulation (taVNS) as a therapeutic tool is worth exploring in the context of consciousness disorders, as previously proposed for invasive forms of VNS, in which the means of stimulating the vagus nerve to change the brain areas related to cosciousness have also received widespread attention. In this paper, we review the literature on taVNS and DOC to better understand the current status and development prospect of taVNS treament as a non-invasive neuromodulation method with sensitivity and/or specificity at the single subject.

## Introduction

Disorders of consciousness (DOC) refer to a persistent state of loss of consciousness that at least four weeks following sudden onset brain injury, including vegetative state/ unresponsive wakefulness syndrome (*VS* /UWS) and minimally conscious state (MCS) (Royal College of Physicians, 2020). *VS*/UWS is an unconscious awakening state in which there is sleep–wake cycles and a range of reflexive and spontaneous behavior. Whereas patients in MCS have emotional and directional behavioral responses such as following instructions, using objects, pain localization, sight tracking, or gazing at the target (Laureys et al., 2010). DOC can also cause a range of debilitating sequelae which require cognitive, motor, communication, emotional, or behavioral rehabilitation of varying intensity and duration. Currently there are about 100,000 new patients with DOC each year in China (Zhao, 2018), and the impact on people’s quality of life and the problems brought to the society are becoming increasingly prominent. Effective treatment are urgent problems to be solved in clinical practice.

Behavioral, pharmacological, and neurostimulatory approaches are the most commonly therapeutic strategies for various neurological diseases (Mishra, 2017). Whether it is neurotransmitter-based drugs or neuromodulation therapy, individual effective case experiences cannot be successfully generalized to the population level (Monti et al., 2015; Sanz et al., 2019; Edlow et al., 2021). Neuroscientists are investigating ways to accelerate the improvement of consciousness in DOC patients while promoting functional recovery. Neuromodulation therapy has shown beneficial effects in promoting recovery after traumatic brain injury, but existing treatments such as transcranial magnetic stimulation, deep brain stimulation (DBS) and hyperbaric oxygen therapy still need further systematic studies to demonstrate their mechanisms of action (Rezaei Haddad et al., 2019; Chen et al., 2022; Huang et al., 2023). The brain function is mediated by the interaction between different neural circuits and neurons in brain regions. The brain, nerves and endocrine systems transmit impulses through neural circuits and regulate internal organ functions. Amantadine is the only intervention recommended by US practice guidelines for DOC (Giacino et al., 2012). Existing approaches all require large, double-blind, randomized controlled trials to confirm possible therapeutic effects. Surgical treatment based on implantation of medical equipment mainly includes invasive brain stimulation such as DBS, which achieve the role of neuroplasticity by sending electrical pulses to specific parts of the central nervous system (Rezaei Haddad et al., 2019). However, the high cost of implantation and the risk of postoperative infection limit its application in clinic to some extent. Therefore, developing new clinical approach that is both effective and safe is urgently needed for the treatment of DOC. Accordingly, we provide an overview of the current parameters, experimental settings and effects of taVNS, and expect the review can contribute to a clear summary of the current research progress of taVNS for DOC.

### Vagus nerve electrical stimulation and neuroplasticity

In terms of the location in the brain where consciousness exists, the more recognised areas are the cerebral cortex, including sensory areas, motor areas and associative areas. The thalamus, situated in the centre of the brain, is implicated in consciousness. Specifically, the thalamocortical loop, entailing the interaction between the thalamus and cortical areas, is deemed crucial for consciousness (Shine et al., 2023). Based on the neurobiology of the vagus nerve and its effect on neural activity, a “bottom-up” therapeutic mechanism has been developed to activate the central nervous system by electrically stimulating the peripheral nerves (Yap et al., 2020). The Vagus nerve provides a major communication channel for brain-integrated, neural reflex regulation of physiological functions (Yuan and Silberstein, 2016). Neuroendocrine-immune network of the vagus nerve involved in maintaining metabolic homeostasis and regulating various pathological processes in the brain (Mravec, 2010)_._

Neurotransmitters or their metabolites, as well as the central nervous system’s function, may be regulated by VNS to alleviate mental disorders (Akhtar et al., 2016). Previous research has established that VNS can effectively regulate motor response and promote motor learning through γ-aminobutyric acid-mediated neuromodulation mechanism (Fitchett et al., 2021). The nucleus tract solitary (NTS) enters the central nervous system and directly projects onto the parabrachial nucleus, thereby projecting to the locus ceruleus (LC) and the dorsal raphe nucleus (DRN) (Krahl et al., 1998). LC is subjected to perpetual stimulation from the prefrontal cortex, which is accountable for executive function. It then transmits to the brainstem, cerebellum, thalamus, hypothalamus, and amygdala. In the hypothalamus, central nervous system (CNS) incitement has been shown to amplify neuronal activity in the PVN, causing the activation of the HPA axis. DRN is the main source of serotonin (5-HT) (Manta et al., 2009). VNS increased the discharge activity of LC and DRN, while regulating basal forebrain activity. These regions are responsible for the release of NE, dopamine (DA), 5-HT and acetylcholine throughout the brain. Using positron emission tomography (PET), the researchers discovered that VNS activated the ventral tegmental area (VTA), which is one of two brain regions responsible for DA release (Val-Laillet et al., 2015). It has been reported that only dopamine metabolites experience an increase in the cerebrospinal fluid (CSF) following VNS therapy (Carpenter et al., 2004). VNS has a positive effect on disorders of consciousness and can regulate the activity of the human brain. The Food and Drug Administration (FDA) approved a prospective randomized cross-over trial to verify that VNS can objectively improve clinical manifestations in patients with severe craniocerebral trauma brain injury (TBI) (Wang et al., 2021). An awakening effect may result from increased cerebral blood flow and metabolic activity of the thalamus and reticular formation in response to the increased cerebral blood flow.

Traditional VNS requires surgery which implant an electrical device in the chest wall next to the cervical branch of the left vagus nerve with the attendant financial costs and risks to the patients (Yang and Phi, 2019). Furthermore, VNS surgery can cause potential adverse events including arrhythmias, hoarseness and other respiratory complications such as cough and nocturnal dyspnoea due to nerve damage, which limits dose regulation in clinical settings and patient tolerance (Goggins et al., 2022). VNS can also cause changes in breathing patterns during sleep, leading to an increase in the number of obstructive apnea and hypoventilation (Gurung et al., 2020). Simultaneously, a battery necessitates replacement every 3–5 years. Furthermore, post-implantation surgery, just 30% of patients exhibit clinical response (Downes et al., 2023).

Non-invasive brain stimulation (NIBS) has the potential to modify the circuit-level of neuronal signalling non-surgically. This can be achieved through manipulation of the relative levels of excitatory and inhibitory signalling, or via activation of the reticular activating system or thalamus-associated nuclei to heighten arousal levels. The procedure is free from side effects that are typically associated with invasive treatments (Zaghi et al., 2009).

### Transcutaneous auricular vagus nerve stimulation (taVNS) in DOC

As a novel and non-invasive neuromodulation method, taVNS achieves therapeutic effects by stimulating the afferent branches of the vagus nerve distributed in the skin. It is discovered that vagus nerve has a branch of afferent projections at the auricular concha (Yuan and Silberstein, 2016). Unlike other non-invasive techniques of brain stimulation, taVNS does not make direct alterations to specific target areas of the cortex’s neurons. Instead, it enhances the noradrenergic neurotransmission by indirectly stimulating the LC and thereby modulates brain functions in a systematic manner (Ruhnau and Zaehle, 2021).

It has been demonstrated that taVNS activated the vagal pathway, which suggests that taVNS is a promising form of VNS (Hilz, 2022; Hilz and Bolz, 2022).Thus, similar effects to those obtained with VNS may be achieved by superficial stimulation of the area in the ear that has vagus nerve innervation. Ventureyra first proposed taVNS in 2000 (Ventureyra, 2000). TaVNS is a non-invasive vagus nerve stimulation method, and its target is the vagus nerve branch of the external ear. After Fallgatter AJ et al. first observed taVNS in 2003 (Fallgatter et al., 2003), repeatable vagus nerve sensory evoking potentials were detected in the scalp, which confirmed that non-invasive vagus nerve stimulation was feasible. Animal studies have shown that taVNS activates cholinergic anti-inflammatory pathways in brain-injured regions, increases Ach levels, inhibits the secretion of inflammatory factors and thus mediates neuroprotection (Zhao et al., 2022).

Although non-randomized controlled trial studies and reviews are not particularly convincing, they do indicate that taVNS may have a role in the emergence from a coma. Yu^[34]^reported a patient in hypoxic encephalopathy treated with taVNS. In just 4 weeks of treatment, the patient’s clinical score increased significantly with intensities of 4-6 mA and a frequency of 20 Hz twice a day. Blood oxygen level-dependent fMRI results indicate obvious activation of the posterior cingulate/anterior gyrus and thalamus, which play a vital role in awakening. Stefan Dietrich and colleagues demonstrated that taVNS can activate the left locus coeruleus, thalamus, left prefrontal cortex, postcentral gyrus, cingulate gyrus, left insular lobe, and other brain regions related to the vagus pathway (Dietrich et al., 2008). A recent fMRI study examining taVNS found that taVNS requires intact auditory function, and can enhance the response to auditory stimuli in patients with DOC (Yu et al., 2021). Potential side effects of taVNS on the brain include tingling caused by electrode stimulation and mild facial twitching with high intensity stimulation. In summary, taVNS is comparable to implanted vagus nerve stimulation, and several studies have demonstrated its safety, feasibility, and efficacy as a treatment option for patients with DOC (Hakon et al., 2020; Noé et al., 2020; Yifei et al., 2022).

### The possible mechanisms of taVNS in DOC

Research into activating vagus nerve stimulation to promote arousal is currently at the feasibility stage (Table 1): Existing studies have gained validity through changes in functional brain networks, UWS/*VS* patients improve with MCS and show improved brain connectivity patterns. A suggested vagocortical pathway model, based on the mechanism of consciousness recovery, proposes that taVNS could offer a therapeutic benefit to patients with DOC by activating brainstem pathways associated with noradrenaline and serotonin (Briand et al., 2020).

**Table 1:** Summary of studies on transcutaneous auricular vagus nerve stimulation in disorders of consciousness.

Authors	Patients	Brain pathology	Disease Duration			Data cycle			Clinical Results	Stimulation parameter			Brain evaluation
			acute	subacute	prolonged	Min/session	Time/d	Period		Hz	Pulse width (us)	Intensity (mA)	
Yu et al. (2017)	1	Anoxia	0	1	0	30	2	50 days	CRS-R: 6 → 13	20	1,000	4 ~ 6	fMRI
Noé et al. (2020)	14	TBI: 7 Anoxia: 4 Hemorrhage: 3	0	0	14	30	2	28 days	CRS-R: 62.5% MCS patients improved	20	250	1.5	-
Hakon et al. (2020)	5	TBI	0	3	1	240	1	56 days	All patients improves	25	250	0.5 ~ 1	-
Yu et al. (2021)	10	Anoxia: 5 Hemorrhage: 3 TBI: 2	2	4	4	30	2	28 days	Responded to auditory stimuli patients get improved	20	500	4 ~ 6	fMRI
Yifei et al. (2022)	12	Stroke: 8 Anoxia: 2 TBI: 2	0	0	12	30	2	14 days	CRS-R: no change	20	1,000	4 ~ 6	EEG

CBF, cerebral blood flow, CRS-R, Coma Recovery Scale-Revised, EEG, electroencephalography, fMRI, functional magnetic resonance imaging, DMN, default mode network, MCS, minimally consciousness state, NC, not commented, TBI, traumatic brain injury, taVNS, transcutaneous auricular vagus nerve stimulation, VS, vegetative state.

Specifically, transcutaneous auricular vagus nerve stimulation results in the activation of NTS by stimulating the trigeminal spinal nucleus, which subsequently activates NTS. Activation of the associated nuclei promotes the release of NE (Zaehle and Krauel, 2021). Evidence suggests that NE is an important mediator of arousal (Ricci et al., 2020). Increasing the concentration of NE in the brain can promote the functional recovery after severe craniocerebral injury. Whether in coma, *VS* or MCS, the increase of DA and NE can improve the level of consciousness (Fukabori et al., 2020). A pooled mega-analysis confirmed that taVNS on salivary alpha-amylase as an indirect marker of noradrenergic activity (Giraudier et al., 2022). The activation of the LC-NE system may be the central mechanism of action of taVNS. The hypothesis that taVNS can enhance central NE release is supported by P300 event-related potentials, which serve as electrophysiological markers of the LC-NE system. A recent study found that taVNS caused pupil dilation and a concomitant decrease in occipital alpha activity in healthy individuals, indicating that taVNS may promote NE release and enhance attention (Chmielewski et al., 2017). Studies have consistently shown that reductions in central norepinephrine have a negative effect on attention. Conversely, increasing NE levels has shown to improve attention, affirming that NE facilitates the function of cortical circuits linked with alertness and attention (Smith and Nutt, 1996; Aston-Jones and Cohen, 2005).

TaVNS has been demonstrated to regulate attention and cognitive performance in healthy individuals, as well as through NTS activation, which may refers to cause changes in the concentration of neurotransmitters such as gamma-aminobutyric acid (GABA) through afferent vagal fiber activation of LC, thereby improving cognition, among other things (Van Leusden et al., 2015; Fischer et al., 2018). Capone et al. proposed that taVNS regulates cortical excitability in healthy subjects by regulating the GABA inhibitory loop (Capone et al., 2015). Amantadine treatment accelerates functional recovery in patients with post-traumatic disorders of consciousness, which may also involved in GABA neurotransmission system (Giacino et al., 2012). The plasticity of the cerebral cortex enables the healthy cortical area to take over some of the functions lost in the damaged area, and the mechanisms that regulate cortical plasticity are shared between the sensory and motor cortex. TaVNS stimulates Aβ-fibers to produce signal pulses that travel from the periphery to the brainstem nuclei and ultimately reach the cortex (Butt et al., 2020).

Increased cerebral blood flow may provide another mechanism by which taVNS operates. It is widely recognized that the rehabilitation of consciousness is correlated with the regeneration of the thalamus cortex (Laureys, 2005). fMRI results demonstrate that taVNS induces significant blood oxygen level-dependent (BOLD) signal changes in the prefrontal cortex of healthy participants. The signal intensity of the prefrontal lobe, thalamus, amygdala, and posterior cingulate gyrus was found to be elevated in the taVNS stimulation group, compared to the control group (Peng et al., 2018). The thalamus selectively transmits information to various parts of the cortex, which is closely related to sleep regulation and consciousness and even plays a key role in regulating wakefulness. Previous studies have found that emotional stimulation can activate the posterior cingulate cortex, which is part of the limbic system and mediates processes related to emotions and memory processing (Maddock et al., 2003).

It has also been suggested that preventing the inflammatory surge after a TBI may prevent systemic inflammatory response syndrome, sepsis, and multi-system organ failure (Johnson and Wilson, 2018). It may also be possible for taVNS to alleviate damages caused by TBI due to widespread neural inflammation. Regulation of cytokine expression by taVNS may provide significant therapeutic value in DOC. There is accumulating evidence to suggest that it can be used to help quell inflammation in a number of other autonomic or inflammatory disorders, which would make it useful for a wider range of patients suffering from disorders of conciousness as well (Falvey et al., 2022).

The enhanced basal metabolic level of the thalamus results in increased CBF in the cerebral cortex, encompassing the somatosensory cortex in the occipital lobe, superior temporal gyrus, and middle temporal gyrus, along with the executive control cortex in the prefrontal region via the intersensory system’s ascending pathway (Rutecki, 1990; Craig, 2003). Simultaneously, enhancing the basal metabolic rate in the insula results in increased metabolism in the somatosensory cortex and prefrontal cortex, both of which play a role in the interoceptive system (Bourdillon et al., 2019). The interoceptive system plays a vital role in preserving a dynamic equilibrium in the body and potentially enhancing self-awareness, the foundation of human emotional well-being and consciousness. Among a variety of sensory pathways connecting to the brain, the vagus nerve is one of them. Consequently, taVNS might elicit the recovery of consciousness by activating the interoceptive system.

Neuroregulation is a physiological process that occurs in normal human life and involves changes in neurons and synaptic properties caused by neurons or substances they release. Nerve stimulation has ability to regulate how the central nervous system processes information, acting as a compensatory mechanism for the loss of normal function caused by disease or injury. While taVNS may not be recommended as a primary or solitary treatment for DOC, existing clinical data suggests that it may enhance brain plasticity and connectivity between the thalamus and cortical region associated with consciousness, thus potentially offering benefits for addressing consciousness disorders.

### Limitations and prospects

Although much progress has been made in the treatment of DOC in recent years, there are still many problems to be solved in diagnosis, prognosis, treatment and rehabilitation of DOC. Patients may receive multiple stimulation methods or drug interventions simultaneously, so large-scale multicenter randomized controlled trials are lacking. The methodological quality of existing studies is low, such as limited simple numbers, high drop-out rates, and lacked proper randomization controls. Further high-quality evidence for the efficacy and safety of taVNS in a multicentre-trial to collect data with adequate sample size, and proper control trials is required. DOC have large differences in etiology and clinical manifestations, which makes it difficult to implement placebo controlled trials. Completely different lesions in the central nervous system can lead to the same clinical manifestations and limit the clinical inclusion criteria^.^ Case reports of non-invasive brain stimulation techniques may be related to natural recovery or other treatments, to some extent, the effect of taVNS on awaking can be seen here. Due to the lack of standardization of methods and the diversity of experimental design, the improvement in clinical behavior is not enough to produce diagnostic changes in the revised version of the coma recovery scale, which may be related to the baseline differences of patients and small sample size. It may also be the source of biases in the results of existing studies, which makes it difficult to interpret the results, so further studies are needed to fully elucidate the mechanistic actions that explain potential role.

In the future, studies may focus on how stimulation affects brain networks and the possible mechanisms involved. It is necessary to design a more rigorous large sample randomized controlled trial, and classify the etiology, course of disease and level of consciousness of the patients in order to determine and verify the effectiveness of the treatment. Researchers are advised to summarize the clinical characteristics of patients who can benefit from the treatment, constantly improve the inclusion and exclusion criteria of the study and analyze cases of adverse reactions. There are many studies that provide inconsistent results on the efficacy of taVNS, either no effect or even the opposite effect. One reason for this may be the variability of the taVNS yield-enhancing parameters in use. There are many different stimulation frequencies (0.5–30 Hz), pulse widths (50-500 μs), intensities (0.5–50 mA) and stimulation positions. Few studies have aimed to assess the role of taVNS parameters on efficacy. This is not only a problem for taVNS, but also for the entire field of neuromodulation. They may do in-depth study of the therapeutic mechanism and constantly optimize the stimulation parameters such as stimulation target, stimulation frequency and stimulation duration to achieve more diversified and lasting clinical improvement.

Therefore, by combining various imaging techniques such as neuroimaging and neurophysiological assessment, we can better grasp the internal anatomical structure and network of the brain to determine the brain area mainly affected by the stimulation method. Neuromodulation and brain-computer interface technologies are cutting-edge hot development directions for future clinical research. According to the residual brain structure and function, we can better determine the beneficiary population and improve the curative effect. It also may be possible to link other physiological parameters associated with vagal stimulation to stimulation efficacy. Among these, the efficacy of taVNS in regulating heart rate and its coupling with neural activity deserves further investigation.

In addition, it has not been proven that taVNS is the decisive cause of changes in consciousness-related brain regions. Other stimulation methods apart from the vagus nerve may also be the causes of the observed results. Therefore, in basic experiments, it is necessary to further clarify the pathological basis of consciousness disorder, explore the physiological basis of the efficacy of taVNS, find suitable biomarkers for taVNS, metabolomics technology may be a biomarker to explore the response of taVNS. If patients can be detected by simple body fluids (blood, cerebrospinal fluid, urine, etc.), it will be an important breakthrough in precision diagnosis and treatment technology. There is an urgent need to seek responsive biomarkers to provide personalized treatment based on the patient’s clinical characteristics and brain lesions. By the way, we may need optimized stimulation parameters such as pulse width, frequency, current intensity, amplitude, and duration to improve treatment efficiency, reduce side effects or adverse reactions, and establish standard procedures in clinical practice.

## Conclusion

Consciousness comes from the brain. The brain can be studied at different but closely related levels, such as genes, proteins, synapses, neurons, neural loops to brain regions and the whole brain. There is no doubt that any single therapeutic approach has its own advantages and limitations and cannot solve all the problems associated with DOC. Meanwhile, any effective means of awakening must not be neglected. Despite clinical and scientific challenges, given that early post-injury period in DOC research is becoming increasingly important, as this is the most critical period for neuroplasticity and medical care decisions that have an undeniable impact on patient survival. Elucidating the mechanisms of consciousness based on existing models of DOC and establishing unique and clinically accurate identification techniques to improve clinical arousal rates remains a daunting task. It is precisely because cost is typically a major factor limiting access to medicines as well as limiting basic research, Ultimately, the potential of taVNS substantially increasing equity in medical care and use of basic science. This review shows that taVNS bears the prospect of being applied to DOC, however, more solid evidences are needed. TaVNS is a promising treatment method, and there are many studies in progress (Table 2). We look forward to the results of these studies can bring new hope to DOC patients.

**Table 2:** Summary of protocal on transcutaneous auricular vagus nerve stimulation in disorders of consciousness.

**Authors**	**Patients**	**taVNS device**	**Data cycle**			**Stimulation parameter**			**Brain** **evaluation**
			**Min/** **session**	**Time/d**	**Period**	**Hz**	**Pulse width** **(us)**	**Intensity** **(mA)**	
Zhai et al. (2023)	15	SDZ-IIB	20	1	NC	1,10,25,50,100	NC	1 ~ 1.5	32- channel EEG
Vitello et al. (2023)	44	NEMOS/tVNS®	45	1	5 days	25	200 ~ 300	3	EEG、EKG
Shou et al. (2023)	90	NC	NC	2	40 days	20 Hz output 7 s, 4 Hz output, and 3 s alternate cycle output	NC	NC	EEG、fMRI
Zhuang et al. (2023)	84	SDZ-IIB	30	2	4 weeks	4 ~ 20	30	1 ~ 1.5	EEG、MRI

CRS-R, Coma Recovery Scale-Revised, EEG, electroencephalography, fMRI, functional magnetic resonance imaging, NC, not commented, taVNS, transcutaneous auricular vagus nerve stimulation.

## Author contributions

YiW: Writing – original draft. JZ: Project administration. WZ: Resources. YuW: Project administration. SL: Project administration. YY: Writing – review & editing. YZ: Resources. JH: Writing – review & editing. PR: Writing – review & editing.
